# 

**Published:** 1921-05-21

**Authors:** 


### The New Architect to Birmingham Hospitals.

Out of a large number of applicants, Mr. D. E. Turner, clerk of works at the Cardiff City Mental Hospital, Whitchurch, has been appointed by the Birmingham Corporation as architect and clerk of works to the three mental hospitals of that city. Prior to his departure, the hospital staff, numbering over one hundred, presented Mr. Turner with an inscribed gold watch. The gathering was presided over by Councillor C. W. Melhuish, who explained that he was not there in his official capacity as Vice-Chairman of the Visiting Committee of the hospital, but as the original employer of Mr. Turner in private life. The gift was handed to the recipient by the medical superintendent, Dr. E. Goodall, C.B.E.; and Dr. Stanford, who added a few words to the tribute paid by Dr. Goodall, said everyone concerned with the mentally afflicted regretted Mr. Turner's departure, and hoped that a worthy successor would be found.

### Medicamentia Recentia.

(Allen and Hanburys', Lombard Street, E.C.)

Under the above title this very old-established firm have issued in compact form and attractive leather binding a booklet setting forth their numerous remedial preparations, with brief notes on their properties, dosage, and usage. This list is really an epitome in little compass of the recent advances in medicine, including vaccine and serotherapy and organo-therapy in general. It would certainly astonish any practitioner who had gone to sleep, let us say, about 1905, and then waked up again, Rip Van Winkle-wise, for he would find that hardly a single condition in medicine is being treated by the ultra-moderns as it used to be. It would be as impolitic to suggest that anyone in the medical profession has actually gone to sleep for the last fifteen years as that there is any human ailment unamenable to vaccines or glandular extracts. Seriously, though, this list of *medicamentia recentia* places the whole profession very definitely in Messrs. Allen and Hanburys' debt.

### Medical Appointments.

Miss Mary Law, M.B., of London, has been appointed assistant schools medical officer under the Newport (Mon.) Education Committee, at a salary of £500 per annum, rising by £25 increments to £600.

Miss E. Dorothy Hawkesworth, M.B., B.Ch., B.A.O. (Hons.), has been appointed assistant school medical officer and assistant medical officer of health to the County Borough of Dewsbury. Miss Hawkesworth is a graduate of Cambridge University.

### Co-operative Buying.

CO-OPERATION is becoming more and more the trend of the day. It is a truism that the energy, brains, business knowledge, and organisation required for the affairs of the individual can be as readily and more profitably employed for the benefit of the many, be they individuals or groups. Co-operative buying has long tickled the fancy of the hospital administrator, and is one of the objects of the peace-time programme of the British Red Cross Joint Council. For schools and other large consumers it has already resolved itself into action, and the recent union of the two organisations, the Schools Co-operative Association, formed in 1920, and the Wholesale Supply Association, known as the Services Central Supply Depôt, should make for a considerable enlargement of the activities of the respective constituents. The new association is to be known as the Schools and Services Supplies, Ltd., and has offices at 16 Coleman Street, London, E.C. The General Manager is Mr. A. E. J. Ferguson (late merchandise manager of Selfridge's), whose business experience should be considerable, and the board of directors includes the names of more than one well-known schoolmaster. Six hundred schools are already among the shareholders in this company (whose prospectus to secure further capital is just issued), and it is evident the services which such a co-operative organisation can render are practically unbounded.

### PASSING EVENTS AND TOPICS.

### Infant Welfare Centres.

The number of such Centres in Melbourne is now numerous. Dr. A. Jeffreys Wood, President of the Victorian Association for the Preservation of Infant Life, states that attendances at the Centres in the different municipaliies for last year totalled 24,892, new babies at the Centres numbered 9,128, nurses had paid 21,240 visits to homes, and 480 expectant mothers were advised. The first Centre was established at Richmond in 1917.

### Pound Day at King's College Hospital.

Pound Day at King's College Hospital, presided over by the Mayoress of Camberwell, was a very successful function. In addition to nearly £60 in cash, goods weighing something like 2,000 pounds were received. A pleasing feature was the interest and help of former patients, who manifested keen concern in the progress of the day's events. Among the cash gifts was that very rare coin, a sovereign, indicating that a valuable relic of former days was not considered by the donor too precious to be cast into the coffers of such an institution.

### Practical Gratitude.

A successful concert was recently held in aid of the Walthamstow Hospital, under the patronage of the Lord Mayor, the Bishop of Barking, Sir Stanley Johnson, and Sir John Bethell, in connection with which a letter was received from the Prince of Wales wishing the enterprise every success. This concert was the outcome of the gratitude felt by the parents of a child patient for the attention and devotion of the staff.

### Telephone Employees Endow Hospital Beds.

The South London Hospital for Women, Clapham Common, S.W., has received from the women of the London Telephone Service a further gift of £500, representing the surplus proceeds of the bazaar held in December last, increased by subsequent subscriptions. By the efforts of the women of the London Telephone Service the Hospital Endowment Fund has now benefited to an amount of £2,500. The two beds and a cot endowed by this means will, by special request, bear the name of the Superintendent of this branch of the Service—Miss A. A. Heap.

### An Eyeless Needle.

Mr. H. S. Souttar, head of the surgical unit of the London Hospital, has invented an eyeless needle, in which, in place of an eye, there is a minute hole bored longitudinally in the head. An end of the catgut can thus be inserted and sealed up, enabling the surgeon to sew with a single thread.

### Great Northern Hospital Bazaar.

Princess Alice, Countess of Athlone, has kindly consented to open the Crewe House Bazaar (in aid of the Great Northern Hospital) on Thursday, June 2, at 3 p.m. The League of the Roses, which has done so much good work for the hospital, will have five stalls, presided over respectively by Mrs. R. Wortley, Jun., Mrs. Cooksey, Miss Garrett, Mrs. Rogulski, and Mrs. E. S. Daniells. All who can are asked to send gifts towards furnishing the stalls.

### Public Health Conferences.

The London Conferences of the Royal Institute of Public Health (offices, 37 Russell Square, W.C. 1) will be held at the Guildhall on Thursday, June 2, to Saturday, June 4. The Duke of York will open the first day's Conference. The Thursday morning session, over which Lord Burnham will preside, will be devoted to "Municipal Hygiene and its Suggested Connection with the Administration of the Poor-law and the Future of Voluntary Hospitals," and among the speakers will be Sir Kingsley Wood, M.P., the Hon. W. Ormsby-Gore, M.P., Sir Napier Burnett, Sir Alfred Fripp, Sir Leslie Mackenzie, and Mr. Hd. Woollcombe, Secretary of the Charity Organisation Society. Subsequent sessions will deal with the "Housing Problem" (Chairman, Sir George McCrae), "The Maintenance of Efficiency and Prevention of Ill-Health in Industry" (Chairman, Professor Edgar L. Collins), "Venereal Diseases and their Prevention" (Chairman, the Bishop of Birmingham), and "The Efficiency of the Present Machinery for Dealing with Tuberculosis" (Chairman, Sir R. W. Philip). A reception will be given to the delegates on the Saturday by Lord Leverhulme at The Hill, North-end, Hampstead.

### New Principal of Livingstone College.

The Committee have appointed Dr. Tom Jays as successor to Dr. Loftus E. Wigram, who has resigned on account of ill-health. Dr. Jays has served since October 1919 as Vice-Principal to Livingstone College. He was formerly a missionary of the Church Missionary Society, and has had much experience in many tropical countries, especially in West Africa. As Travelling Secretary of the Student Volunteer Missionary Union he has visited the chief Universities and Colleges of Great Britain and of the United States. By his appointment the continuity of the work of the College will be maintained, as it is hoped still to retain the services of Dr. Wigram as a lecturer.

### Hospitals and Industrial Unrest.

Mr. George Verity, at the annual meeting of Charing Cross Hospital, said that industrial unrest is having a very detrimental effect on hospitals. He looked forward to one of the leanest years the country has ever experienced, in which the balance of £4,579 carried forward at his hospital would be needed. A feature of the report was the remarkable success of the scheme of voluntary payment from patients.

### A Self-supporting Home.

The resources of the Private Nurses' Home of the Hospital for Sick Children, Great Ormond Street, are being severely taxed. It is entirely supported by the earnings of the nurses and has no margin for any unforeseen emergency. Such a one has recently occurred through a serious operation in the American Hospital in Paris on one of the Home's nurses who was on duty in that city. The costs incurred have been very heavy, and it has become obvious that the Home must have additional funds on which it can draw when necessity arises. To provide these a concert was held on May 2 at Steinway Hall, Wigmore Street.

### Industrial Medicine.

The Industrial Welfare Society is holding a conference of industrial medical men on June 2, at 51 Palace Street, Westminster, S.W. 1, when addresses will be given by recognised experts in this branch of medicine. All interested in the subject should apply to Mr. R. R. Hyde, Director, Industrial Welfare Society, 51 Palace Street, Westminster, S.W. 1.

### St. Bernard's Convalescent Home for Invalid Gentlewomen.

This Home, established fifty years ago at Brunswick Place, Hove, is threatened with extinction. The lease of the present premises has expired, and fresh ones have to be sought. It is impossible to find a house to rent, and money for purchase is required. The Marchioness of Carisbrooke, President of the Home, is holding a drawing-room meeting at her house, 4 Belgrave Place, on May 30, and a ball will be given at the Hyde Park Hotel on June 28, at which she will be Receiving Hostess. H.R.H. the Duchess of Albany has already sent a cheque, and it is hoped others will follow suit. The Hon. Organising Appeal Secretary is Miss Erica Beale, 108 Queen's Gate, S.W., and cheques should be made payable to St. Bernard's Home Special Appeal Fund and crossed "London County Westminster and Parr's Bank."

### "Progress."

Miss C. S. Ross, Matron of the British Hospital at Nazareth, contributes an interesting article to the March number of "Progress," the journal of the Great Northern Central Hospital, with which, by the way, the Royal Chest Hospital will soon be combined. The number also contains a letter from the editorial department of "Hospital Management," a Chicago paper, inviting co-operation between "Progress" and itself. The Committee of Management lately passed resolutions in favour of payment in full by approved societies of insured patients in both departments, and of "A Voluntary Hospitals Publicity and Propaganda Committee," to which all hospitals should subscribe. This Committee should consist of hospital representatives and publicity experts, and promote "the interests of voluntary hospitals generally," leaving it to each hospital to make known its own needs.

### Suffolk Nursing Association.

A very satisfactory financial position was revealed at the annual meeting of the Bury St. Edmunds Branch of the Suffolk Nursing Association. The nursing tends to become more and more self-supporting, a decrease in subscriptions being more than counterbalanced by an increase in fees. The British Red Cross Society contributes £30 per nurse. Permanent premises have been acquired for the nurses in a central position, and the wish expressed by the Chairman that the Association should become a thoroughly organised business proposition is in the way to being realised.

### Leeds District Nursing Association.

The forty-second annual meeting was presided over by the Mayor of Leeds and the Lady Mayoress. Considerable progress was reported, particularly in respect to the nursing of influenza patients, undertaken at the request of the Medical Officer of Health. The number of visits paid totalled 71,815, and, in spite of increased expenditure, the Association started the New Year with a balance in hand. Close on £2,000 was received in small contributions from working-class people.

### Presentation to Matron on Retirement.

Miss Willans, who has lately retired from the office of matron at the Weybridge Cottage Hospital, which she has held for fifteen years, has been presented with a cheque for £154 as a testimonial from numerous friends and old patients. The institution flourished under her capable management, and there is widespread regret at her departure.

### Nursing Progress in the West Riding.

The proceedings at the recent meeting of the West Riding Nursing Association in Leeds, under the chairmanship of the Lord Mayor of that city, testifies to a live spirit in Yorkshire nursing circles which is very refreshing. There are now sixty-five affiliated associations, employing seventy-two nurses, of whom twenty-five were trained by the Central Association. An interesting feature is the attendance of 1,507 patients at the Nurses' Home. Mrs. Kitson Clark, endorsing the Lord Mayor's eulogies on the nurses, declared no one could measure the value of the work of the nurses—it was some of the finest work being done in England.

### Forthcoming Events.

The Lebanon Hospital (for Mental Diseases), Asfuriyeh, near Beyrout, Syria (London Office, 35 Queen Victoria Street, E.C.).—Annual General Meeting, 19 Russell Square, W.C. 1, Wednesday, May 25, at 3 p.m. Tea at 4.30.

A meeting will be held in the Nurses' Club, 8 Drumsheugh Gardens, Edinburgh, on Wednesday, May 25, at 3.30 p.m., when Miss Honeyburn (London) will speak on "Purity Work."

### Queen Mary's Hospital, Stratford.

Through the generosity and good work of the South-West Ham Committee, this hospital is now in possession of its own Convalescent Home at Theydon Towers. The Home, which was used as an auxiliary hospital during the war, was opened at the beginning of May by H.R.H. Princess Mary; it has accommodation for thirty children, and the South-West Ham Committee, besides giving the Home and grounds, is attempting its maintenance and endowment. The success which has attended the Committee's efforts since its formation in 1917 (it has raised over £8,700) warrants the belief that it will succeed in this attempt.

### The Unmarried Mother and her Child.

At the invitation of Mrs. Scharlieb, an "At Home" was given at 149 Harley Street, on May 12, to further the aims and explain the needs of the National Council for the Unmarried Mother and her Child. Mrs. H. A. L. Fisher eloquently presented the Council's work, and Capt. G. E. W. Bowyer, M.C., M.P., gave a brief summary of the Bastardy Bill which is still awaiting its second reading in the House of Commons. The effect of its provisions, not the least important of which is the increase of the sum payable under an affiliation order from 10s. to 40s., was dealt with in our issue of April 30. Though it is an agreed Bill, and is practically the same as when it came out of Committee last year, when Mr. Neville Chamberlain piloted it through the Commons, it has met with a certain amount of quiet blocking, and Captain Bowyer has found that ardent advocacy, even when combined with the spirit of compromise, is no match for tradition, Parliamentary experience, and an unsurpassed knowledge of procedure. The aim of the National Council for the Unmarried Mother, whose Bill this is, is to keep the mother and her child together. This is recognised as the key to the salvation of the child; and the record of the work done by this Council, first at Evelyn House, 62 Oxford Street, and now at 20 Berkeley Street, W. 1, shows the wide field there is for such a centre of information, advice, and propaganda.

### The Nursing and Midwifery Exhibition.

The interest which this Exhibition excites each year cannot fail to surprise even its most enthusiastic supporters. This is not written from any disrespect to the eleventh annual event, which was opened on Tuesday, May 17, at the Horticultural Hall, and which is as excellent as any of its predecessors. Why should nurses and midwives, who are constantly surrounded by much that the Exhibition shows, and more or less live in the atmosphere created by it, come regularly year after year in their off-duty hours to visit the various stalls and examine the various articles displayed? The answer can only reflect the greatest credit both on the visitors and on the Exhibition organisers. This year, despite the general industrial difficulties and the fact that the Exhibition opened at 12 o'clock on the day following Whit Monday, the stalls were all practically complete when we visited it early on the first afternoon. The palm for the greatest originality in stall decoration may, we think, be claimed by A. Wander, Ltd., whose scheme, with its futurist drawings, is intended to represent the Blue Bird of Maeterlinck, which in this case was Health, to be gained by use of the firm's various productions, such as Ovaltine, Cristolax, and the Formalin products. Less subtle but very attractive to the eye is the Allenbury stall, really almost a saloon, so roomy and charmingly arranged is it. And so on throughout the Exhibition, each stall has its distinctive scheme of decoration, in some cases related to the articles displayed, in others merely to please and attract. With the band playing in the gallery, a restaurant on the premises, and a "Glaxo" Welcome room, to say nothing of innumerable free samples of tooth-paste, chocolates, perfume, suet, etc., from many stalls, and a most attractive exhibit of the Gas Light and Coke Co., in the shape of a combined kitchen, scullery, and larder, which includes kitchen stove, washing-up sink, saucepan rack, and other details, the standard size of the whole cabinet being 7 ft. by 4 ft. by 2 ft. 6 in., the industrious nurse seems to have here all she requires for the provision of an interesting and pleasant afternoon. The Conferences on various subjects which continue throughout the last three days are always well attended, and play their part, too, in attracting.

Hospitals in Liverpool—namely, the Royal Infirmary (£100), the David Lewis Northern Hospital (£50), and the Royal Southern Hospital (£50)—benefit under the will of Mr. John Roberts, Liverpool.

### THE COLLEGE OF NURSING (Limited by Guarantee).

### BIRMINGHAM THREE COUNTIES CENTRE.

(Hon. Secretary, Mrs. Glegg, 84 Hagley Road.)

On May 12 Dr. T. C. Graves, M.D., F.R.C.S. (Eng.), medical superintendent of Rubery Mental Hospital, Birmingham, gave an address on "The Position of a Knowledge of Mental Diseases in the General Nursing Curriculum." Dr. Graves commented on the fact that an acquisition of knowledge concerning mental diseases is obligatory on general medical practitioners, whereas in the curriculum of general nursing it has been assigned no position. He believes the present a favourable time to consider the desirability of remedying this omission and of bridging the hiatus between the two branches of nursing. Dr. Graves presented three questions for the consideration of his audience: (1) Why has a fully trained nurse no knowledge of mental diseases? Because the realisation that a disordered mind is a condition of illness is a product of modern times. It was not until the beginning of the nineteenth century that the physiological conception of insanity was accepted and the study of the anatomy and physiology of the brain became general. (2) Is the knowledge of mental diseases desirable in general training? Owing to the extended scope of nursing, yes. Gynæcology and psychology are closely related, and insanity may occur at any time during pregnancy. A knowledge of mental diseases would be of incalculable value to nurses engaged in preventive medicine and to nurses in private practice. (3) How can the knowledge be obtained? Three months out of a general training should be allocated to mental work; the ideal scheme is a clinic for nervous diseases, with an in-patient and out-patient department, under the auspices of a general hospital, where incipient mental cases can obtain treatment.

### NORFOLK AND NORWICH CENTRE.

(Hon. Secretary, Miss V. A. Rutter, Norfolk and Norwich Hospital.)

The annual meeting of the Centre was held at the Norfolk and Norwich Hospital on Tuesday, April 26, 1921, Miss Cann, R.R.C., in the chair. Miss Thorp, Lady Superintendent, Norfolk and Norwich Staff of Nurses, was elected Chairman for the coming year, and the Hon. Secretary and Hon. Treasurer were re-elected. The membership is seventy, with the promise of many more members for the coming year. Lady Gurney gave an interesting address on "Nursing from an Outsider's Point of View."

### READING CENTRE.

The adjourned meeting of the Reading Centre will be held on Wednesday, May 25, at 3 p.m., at the Royal Berkshire Hospital.

### SHEFFIELD CENTRE.

(Hon. Secretary, Mrs. J. Barnes, 34 Wilkinson Street.)

Miss Sheriff MacGregor addressed a meeting of the members on May 2. At the outset of her remarks she emphasised the need of always remembering that the members were the College, and that the Local Centre was the College of Nursing in the particular place in which such Centre was established. Only by efforts of the individual members could the success of the College as a whole be assured. The speaker then dealt with the various activities of the College, such as the raising of the salaries of nurses in all spheres of their work, the establishment of a Chair of Nursing, the granting of Scholarships, the opening of a country cottage at Bonchurch for the benefit primarily of nurses convalescing after illness, and then for holiday purposes. She also explained at some length the work of the Tribute Fund for Nurses. Another need is the establishment of an endowment assurance fund and a superannuation fund for nurses.

### COUNCIL ELECTIONS.

Miss Janet W. Macdonald writes from the London School of Economics and Political Science that she is withdrawing from nomination as a candidate for the College of Nursing Council, as she has been offered an appointment in India, which combines to a certain extent nursing with social welfare.

### Matrons' and Sisters' Appointments.

Tuberculosis Sanatorium, Rushden (Northants County Council).—Miss Beatrice Allsop has been appointed matron. She was trained at St. Thomas's Hospital, and has been sub-matron of the Royal Sea Bathing Hospital, Margate.

Royal Albert Edward Infirmary and Dispensary, Wigan.—Miss Agnes Tinsdeall has been appointed night sister. She was trained at the Manchester Royal Infirmary and Bradford City Hospital; has been night sister at the latter hospital and at the Birmingham City Hospital.

Westmorland Sanatorium for Men, Grange-over-Sands.—Miss Jessy Mellows has been appointed sister. She was trained at Brompton Hospital and St. Marylebone Infirmary; she has been sister of the military wards at Torbay Hospital, night sister at Bury Infirmary, and sister of medical and surgical wards at the East Suffolk Hospital, Ipswich. She has done private nursing, and is a member of the College of Nursing.

Rosedale Hospital, Dumfries.—Miss Emily Morgan has been appointed matron. She was trained at Newcastle-on-Tyne Royal Infirmary, where she was later sister and night superintendent. She has been successively matron of Wall-send-on-Tyne, Crewe, and Harrogate Borough Hospitals. She took her maternity training at Glasgow Maternity Hospital.

Isolation Hospital, Winchmore Hill.—Miss Florence H. Cobham has been appointed night sister. She was trained at the London Temperance Hospital and the Isolation Hospital, Winchmore Hill. She has been ward sister at the London Fever Hospital, the West Middlesex Hospital, and at the Southern Hospital, Dartford.

Birkenhead Union Infirmary.—Miss Edith S. Meakin has been appointed night superintendent nurse. She was trained at Toxteth Park Poor Law Institution, where she was later ward sister. She has been health visitor and school nurse under the St. Helen's Corporation, and ward sister at the Birkenhead Union Infirmary.

Royal Westminster Ophthalmic Hospital.—Miss L. Richards has been appointed matron. She was trained at the General Eye Hospital, Swansea, where she was later sister-in-charge. She has been ward sister of the Royal Eye Hospital, Manchester, and assistant matron of the Royal Infirmary, Gloucester.

Isolation Hospital, Altrincham.—Miss Kathleen Maun has been appointed senior sister. She was trained at the North Devon General Hospital, Barnstaple, and has since been staff nurse at the Coronation Hospital, Ilkley, Yorkshire.

Royal Hospital, Richmond, Surrey.—Miss Eleanor Watt has been appointed matron and superintendent of nurses. She was trained at the Prince of Wales's General Hospital, and from 1917 to 1918 served at the General Military Hospital, Edmonton, then becoming night superintendent at the Royal Hospital, Richmond, and later, after an interval, as ward sister at the Prince of Wales's General Hospital. During the late matron's illness Miss Watt was appointed acting matron. Miss Norah K. Vere has been appointed assistant matron. She was trained at the Seamen's Hospital, Greenwich, and the Women's Hospital, Soho. She was appointed ward sister at the Royal Hospital, Richmond, in 1918, and left to take up nursing in the south of France. She returned to Richmond, on the invitation of the late matron, in May last as senior sister.

Blencathra Sanatorium, Threlkeld.—Miss G. Wyrill has been appointed matron. She was trained at St. Pancras Infirmary, and has had considerable experience in sanatoria. Her present post is matron of Preston Borough Sanatorium.

Royal Hospital for Sick Children, Aberdeen.—Miss M. G. Gould has been appointed night sister. She was trained at the Royal Infirmary, Leicester, and the Royal Hospital for Sick Children, Aberdeen.

Hospital for Women, Shaw Street, Liverpool.—Miss Eva M. Manning has been appointed lady superintendent. She was trained at the Royal National Hospital, Ventnor, the Samaritan Hospital for Women, Marylebone, and the Hospital for Women, Shaw Street. She is at present matron of the Jones Convalescent Home, Minera, near Wrexham.

Brecknock County and Borough Infirmary.—Mrs. M. Bailey has been appointed matron. She was trained at Worcester General Infirmary, and after various charge posts is lady superintendent of the District Nurses' Home, Wednesbury, Staffs.

### Answers to Correspondents.

Homes for Chronic Cases.—You do not say whether you require a home for a man or woman. Full particulars are necessary if you wish us to help you. There are such homes as you refer to, and you will find a list in Burdett's "Hospitals and Charities," a copy of which can be seen at most public libraries.—C. L.

Midwifery Training.—We suggest that you apply to one of the following institutions, where you can get the training you require: The Rotunda Lying-in Hospital, Dublin, and the National Maternity Hospital, Dublin. Address your application in each case to the Superintendent. We advise you to obtain the book "How to Become a Nurse," published by The Scientific Press, Ltd., 28 and 29 Southampton Street, Strand, W.C., price 2s. 10d. post free. In this you will find some useful information upon this subject. We regret we cannot give postal replies. A coupon should be enclosed with each inquiry.—N. M.

**RATES OF SUBSCRIPTION** (post free, payable in advance).

Three months, 3/3; six months, 6/6; twelve months, 13/-. (Should the cost of printing and paper as well as increased postage, necessitate alteration of these rates, the period paid for will be reduced proportionately on unexpired subscriptions.) THE HOSPITAL "Index" to Volume LXIX., October 1920 to March 1921, is now ready, price 1/1 per copy, post free. Address The Manager, The Hospital, "The Hospital" Building, 28/29 Southampton Street, Strand, London, W.C. 2.

